# Dioxane-Linked Novel Bacterial Topoisomerase Inhibitors Exhibit Bactericidal Activity against Planktonic and Biofilm Staphylococcus aureus *In Vitro*

**DOI:** 10.1128/spectrum.02056-22

**Published:** 2022-10-17

**Authors:** Anna Chen, Sheri Dellos-Nolan, Yanran Lu, Jason S. West, Daniel J. Wozniak, Mark J. Mitton-Fry

**Affiliations:** a Microbial Infection and Immunity, College of Medicine, The Ohio State University, Columbus, Ohio, USA; b Division of Medicinal Chemistry and Pharmacognosy, College of Pharmacy, The Ohio State University, Columbus, Ohio, USA; c Department of Microbiology, College of Arts and Sciences, The Ohio State University, Columbus, Ohio, USA; Defence Science and Technology Laboratory

**Keywords:** biofilm, *Staphylococcus*, topoisomerase

## Abstract

The development of novel treatments for Staphylococcus aureus infections remains a high priority worldwide. We previously reported compounds 0147 and 0186, novel bacterial topoisomerase inhibitors (NBTIs) with potent antibacterial activity against S. aureus, including methicillin-resistant S. aureus. Here, we further investigated the *in vitro* activity of 0147 and 0186 against S. aureus ATCC 29213. Both compounds demonstrated bactericidal activity against planktonic and biofilm S. aureus, which then translated into significant inhibition of biofilm formation. Combinations of NBTIs and glycopeptides yielded indifferent interactions against planktonic S. aureus, but several had synergistic effects against S. aureus biofilms. This work reinforces the potential of NBTIs as future therapeutics for S. aureus infections.

**IMPORTANCE** The pathogen Staphylococcus aureus contributes substantially to infection-related mortality. Biofilms render bacteria more recalcitrant to antibacterial therapy. The manuscript describes the potent activity of a new class of antibacterial agents against both planktonic and biofilm populations of Staphylococcus aureus.

## INTRODUCTION

Staphylococcus aureus, a Gram-positive, opportunistic pathogen, remains a serious concern worldwide. It has been shown to cause both community-acquired and nosocomial infections, including bacteremia, skin and soft tissue infections, infective endocarditis, and medical device-related infections. (1, 2). S. aureus also readily forms biofilms, aggregates of bacteria embedded in an extracellular matrix that are recalcitrant to antibiotics and highly difficult to treat (3). This is clinically relevant for medical conditions such as cystic fibrosis, where S. aureus is the predominant microorganism in the lungs of affected children (4).

Infections by both methicillin-susceptible and methicillin-resistant S. aureus (MSSA and MRSA, respectively) are highly problematic. Studies indicate a higher incidence rate for MSSA than MRSA and an increasing number of community-onset MSSA infections in the United States (5, 6). At the same time, antimicrobial resistance (AMR) continues to be a worldwide problem, with approximately 1.27 million deaths in 2019 attributable to bacterial AMR (7). MRSA is among the deadliest pathogens, with more than 100,000 attributable deaths globally in 2019 (7) and 10,600 deaths in the United States in 2017 (8). These findings highlight the seriousness of S. aureus bacterial infections and the rise of resistant strains, and they call for the discovery and development of novel therapeutics.

**FIG 1:**
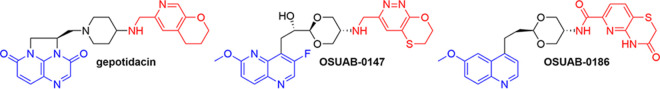
Structures of NBTIs under investigation.

Novel bacterial topoisomerase inhibitors (NBTIs) are a new class of antibacterial agents being explored in the face of rising antibiotic-resistant bacterial pathogens (9, 10). As illustrated by the clinical candidate gepotidacin (Fig. 1), the NBTIs are characterized by a DNA-binding moiety (blue), a central linker (black), and an enzyme-binding group (red) (11). Like fluoroquinolones, NBTIs target DNA gyrase and topoisomerase IV, but their binding and detailed mechanisms of action are distinct, enabling NBTI activity against fluoroquinolone-resistant strains. NBTIs typically induce DNA single-strand breaks, while fluoroquinolones generally induce double-strand breaks (9, 11, 12). Gepotidacin has completed phase 2 clinical trials to treat uncomplicated urinary tract infections (13), urogenital gonorrhea (14), and Gram-positive acute bacterial skin and skin structure infections (15) and is currently in phase 3 clinical development (16). Additionally, Bugworks recently announced the start of phase 1 clinical trials of another NBTI, BWC0977 (ClinicalTrials.gov registration no. NCT05088421). The progress of these NBTIs has showcased the potential of this novel class and has served as a further motivation for our studies.

The current study focuses on the *in vitro* activity of two of our recently developed dioxane-linked NBTIs, amine OSUAB-0147 (Fig. 1; here 0147) (17) and amide OSUAB-0186 (0186) (18) on planktonic and biofilm S. aureus ATCC 29213. We determined MICs, minimum bactericidal concentrations (MBCs), and time-kill kinetics to evaluate their *in vitro* effectiveness against planktonic S. aureus. We also employed inhibition of biofilm formation and biofilm eradication assays to measure the abilities of these NBTIs to inhibit biofilm formation and eradicate mature biofilms, respectively. Last, using both single and combination treatments (with glycopeptides), we investigated the capacity of the compounds to eradicate planktonic and biofilm S. aureus *in vitro*.

## RESULTS

### Compounds 0147 and 0186 demonstrate killing of planktonic S. aureus ATCC 29213.

The MICs and MBCs of 0147, 0186, and comparator agents were determined to establish whether each compound is bactericidal versus bacteriostatic (Table 1). Gepotidacin was found to have an MIC of 0.5 μg/mL, 0147 exhibited an MIC of 0.125 to 0.25 μg/mL, and 0186 demonstrated a lower MIC of 0.0625 μg/mL; all MICs were similar to previously reported values (17–19). MBC values for gepotidacin (0.5 to 1 μg/mL, 1 to 2× MIC), 0147 (0.25 μg/mL, 1 to 2× MIC), and 0186 (0.125 to 0.25 μg/mL, 2 to 4× MIC) demonstrated bactericidal activity for all three NBTIs. Bactericidal (ciprofloxacin, oritavancin, and vancomycin) and bacteriostatic (erythromycin and tetracycline) controls behaved as expected. The MIC values were subsequently used to establish starting concentrations for time-kill assays.

**TABLE 1:** MICs and MBCs of NBTI compounds and comparator agents for S. aureus ATCC 29213^*a*^

Compound	Class	MIC (μg/mL)	MBC (μg/mL)	MBC/MIC	Bactericidal or bacteriostatic
0147	NBTI	0.125–0.25	0.25	1–2	Bactericidal
0186	NBTI	0.0625	0.125–0.25	2–4	Bactericidal
Gepotidacin	NBTI	0.5	0.5–1	1–2	Bactericidal
Ciprofloxacin	Fluoroquinolone	0.25	0.5	2	Bactericidal
Oritavancin	Glycopeptide	2–4	2–4	1	Bactericidal
Vancomycin	Glycopeptide	1–2	2	1–2	Bactericidal
Erythromycin	Macrolide	0.5	4–8	8–16	Bacteriostatic
Tetracycline	Tetracycline	1	>8	>8	Bacteriostatic

a Results represent three independent experiments performed in triplicate.

**FIG 2:**
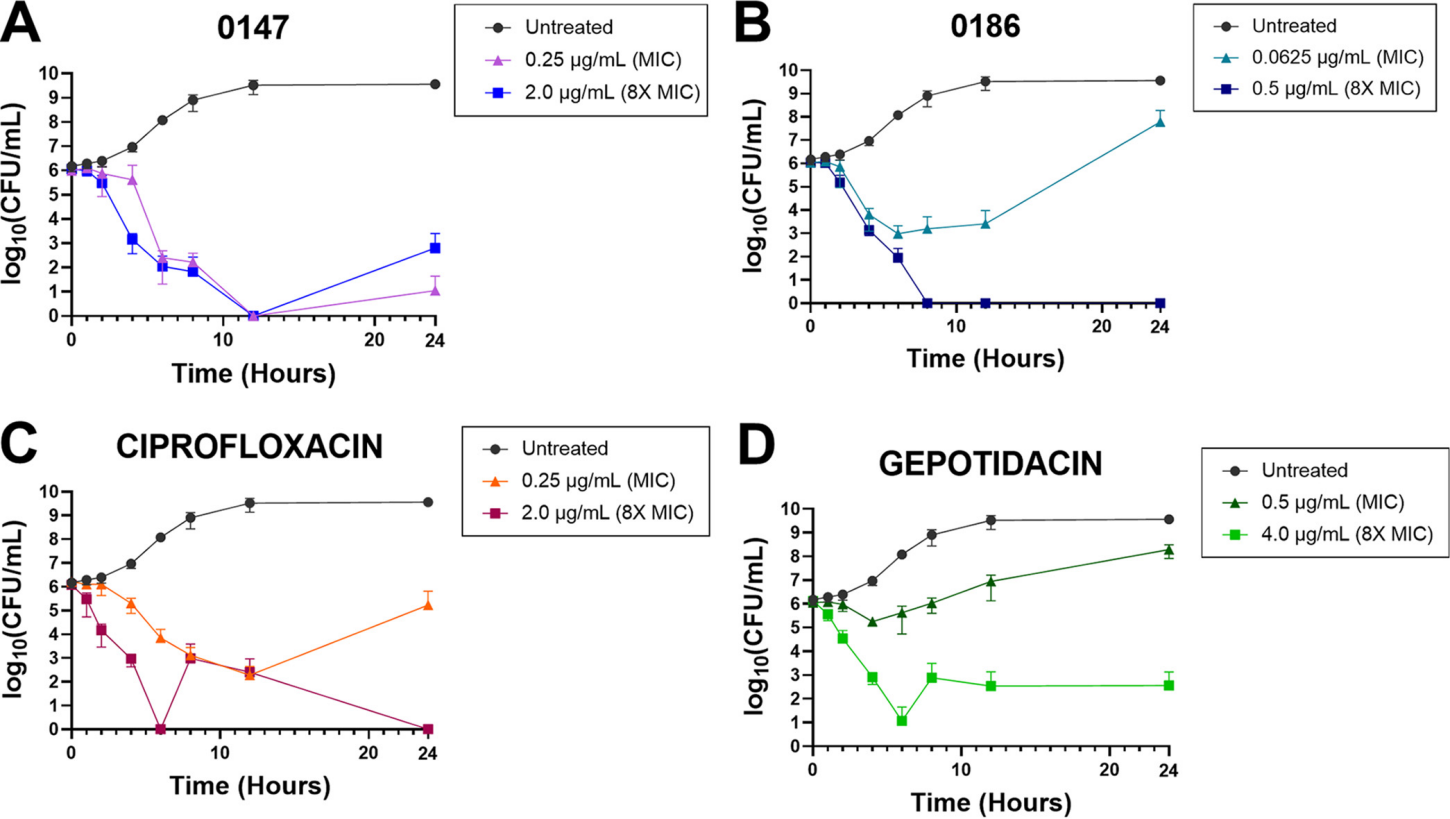
Time-kill kinetics of NBTI compounds and comparator agents for S. aureus ATCC 29213. (A) 0147; (B) 0186; (C) ciprofloxacin; (D) gepotidacin. Untreated time (*T*) of 0 h, 1.67× 10^6^ CFU/mL; untreated *T* of 24 h, 3.66× 10^9^ CFU/mL. Results represent an average of three independent experiments performed in triplicate.

We further investigated the bactericidal kinetics of gepotidacin, 0147, and 0186 using a time-kill assay with planktonic S. aureus 29213 (Fig. 2). The positive control, ciprofloxacin, exhibited bactericidal activity at 8× MIC, with no recoverable bacteria by hour 24. Compound 0147 demonstrated bactericidal activity at both the MIC and 8× MIC. Compound 0186 at the MIC demonstrated a 3-log_10_ decrease before regrowth was observed after hour 6. Compound 0186 was bactericidal at 8× MIC, and CFU were below the limit of detection at 24 h. Gepotidacin was bactericidal at 8× MIC, but regrowth was observed at the MIC, similar to previously reported observations (19). In summary, all three NBTIs demonstrated bactericidal activity at 8× MIC in the time-kill studies (Fig. 2), as has been seen before with gepotidacin and another NBTI reported by D’Atansio et al. (20).

### Compounds 0147 and 0186 prevent biofilm formation.

**FIG 3:**
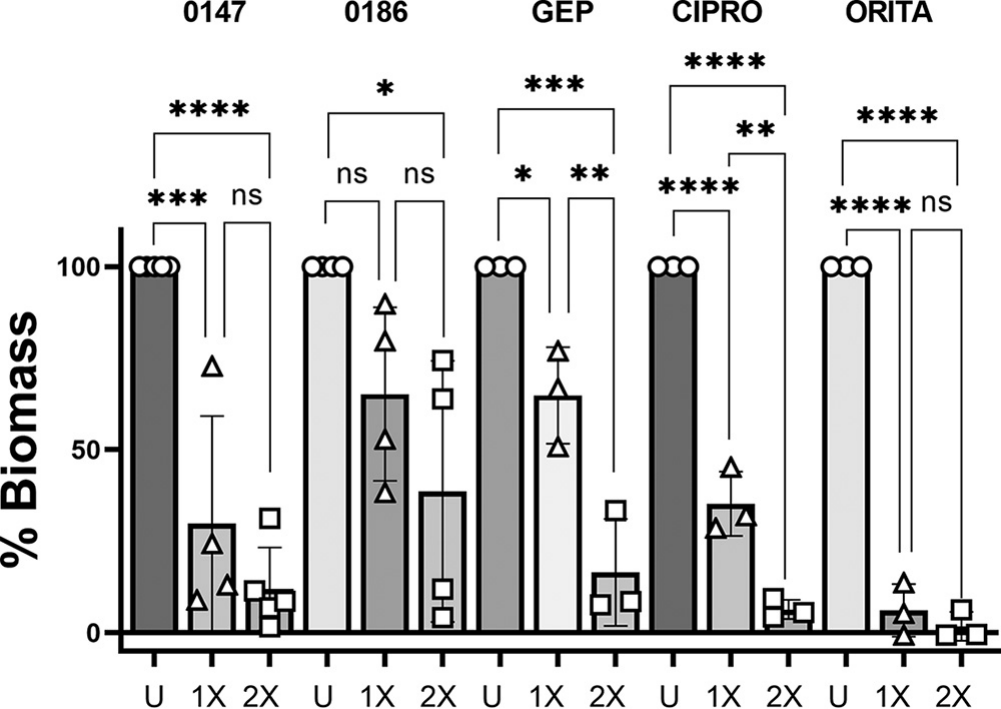
Percentage of S. aureus ATCC 29213 biofilm biomass after treatment by NBTI compounds or comparator agents. GEP, gepotidacin; CIPRO, ciprofloxacin; ORITA, oritavancin; U, untreated; 1×, 1× MIC; 2×, 2× MIC. Results represent an average of three or four independent experiments performed in triplicate. Analyzed by one-way ANOVA (ns, *P* > 0.05; *, *P* < 0.05; **, *P <* 0.01; ***, *P <* 0.001; ****, *P* < 0.0001).

Given the importance of staphylococcal biofilms in clinical settings (21, 22), we evaluated the ability of 0147 and 0186 to prevent biofilm formation. To more readily detect S. aureus biofilm growth, 96-well plates were pretreated with poly-l-lysine, and the Luria broth (LB) growth medium was supplemented with 2% glucose and 2% NaCl (23). Approximately 10^7^ CFU/mL of S. aureus was incubated with the test compound for 24 h at 37°C. Crystal violet was utilized to stain the resulting biofilm biomass and compare it with an untreated control. Due to the antibacterial activity at the concentrations tested, the growth inhibition translated into a concomitant reduction in biofilm formation. In previous studies, oritavancin demonstrated exceptional eradication of stationary-phase and slow-growing S. aureus (24) and therefore was utilized as a positive control for this assay and the following biofilm bead model. Oritavancin at the MIC and 2× MIC demonstrated robust activity, with 94% and 98% growth reductions compared to the untreated control (Fig. 3). Ciprofloxacin exhibited a 65% reduction at the MIC and 94% at 2× MIC (Fig. 3). Gepotidacin and 0147 demonstrated substantial and statistically significant reduction in biofilm growth at either the MIC or 2× MIC, with both achieving similar reductions (84% and 88%, respectively) at 2× MIC (Fig. 3). Compound 0186 achieved statistically significant reduction (61%) only at 2× MIC (Fig. 3). Reduction of biofilm mass was observed to be concentration dependent for some cases described below; however, increased concentrations of antibiotics above 2× MIC generally did not significantly reduce biofilm formation further (data not shown).

### Compounds 0147 and 0186 demonstrate eradication of preformed S. aureus ATCC 29213 biofilms.

We adapted the model described by Konrat et al. based on cultivating biofilm on beads (25). This model has several advantages, including the ability to create more dynamic biofilms *in vitro*, flexibility in adjusting growth conditions, and ease in transporting the beads to different media. In our modified biofilm bead model, S. aureus ATCC 29213 was able to establish a mature biofilm on the polystyrene beads after a 24-h incubation period with an average density of 3.37× 10^7^ CFU/mL. Compounds 0147 and 0186 exhibited MBEC values of 1 μg/mL (4× to 8× MIC) and 4 μg/mL (64× MIC), respectively. Gepotidacin had an MBEC of 8 μg/mL (16× MIC), ciprofloxacin had an MBEC of 1 μg/mL (4× MIC), and oritavancin had a MBEC of 8 μg/mL (2× to 4× MIC) (Table 2). The high MBEC/MIC ratio for 0186 could be a result of reduced biofilm penetration or reduced susceptibility toward slow-growing and stationary-phase S. aureus (26); however, both 0147 and 0186 demonstrated eradication of S. aureus biofilm at low MBEC values, providing encouraging results toward future biofilm treatment.

**TABLE 2:** MBECs of NBTI compounds and comparator agents for Staphylococcus aureus ATCC 29213^*a*^

Compound or drug	MBEC (μg/mL)	MBEC/MIC
0147	1	4–8
0186	4	64
Ciprofloxacin	1	4
Gepotidacin	8	16
Oritavancin	8	2–4
Vancomycin	4	2–4

a Results represent an average of three to seven independent experiments performed in triplicate.

### Compounds 0147 and 0186 exhibit synergistic effects with certain glycopeptides against S. aureus biofilm.

Combination therapy is one strategy being utilized against hard-to-treat biofilms (27). We hypothesized that NBTI biofilm activity could be enhanced if combined with existing antibiotics that have been shown to impact biofilm-grown cells (22, 27). Vancomycin is currently commonly used to treat clinical MRSA infections and has shown promise in combination treatments (22, 28–30), while oritavancin has been shown to eradicate stationary-phase and biofilm S. aureus (24, 31). As a result, we chose the glycopeptides oritavancin and vancomycin for our combination treatments. We tested various sub-MBEC concentrations of 0147 and 0186 paired with vancomycin or oritavancin. We determined the combination MBEC values of the NBTIs and glycopeptides with the same standards (99.9% or 3-log_10_ reduction in CFU/mL) as the single treatment. From these data, the fractional biofilm inhibitory concentration (FBIC) was calculated to determine potential synergistic relationships (Table 3). The combinations of 0147 and vancomycin, 0186 and vancomycin, and 0186 and oritavancin were synergistic, yielding FBIC values of 0.5, 0.5, and 0.3125, respectively. The other combination treatments, however, were indifferent against S. aureus biofilm. The potential for synergistic combinations was also evaluated for planktonic S. aureus via the checkerboard assay. We found that these same combinations on planktonic bacteria yielded indifferent results (Table 4). Ultimately, the most promising synergistic effects on S. aureus biofilm were the combinations of 0147 and vancomycin, 0186 and vancomycin, and 0186 and oritavancin. These results illustrate the potential of combining NBTIs with other antibiotics to treat S. aureus biofilms.

**TABLE 3:** Fractional biofilm inhibitory concentration (FBIC) analysis for NBTI combination treatments^*a*^

Drug or compound	MBEC (μg/mL), alone	MBEC (μg/mL), combination	FBIC value (interpretation)
0147	1	0.25	
Vancomycin	4	1	
0147 and vancomycin			0.5 (synergy)
0147	1	0.5	
Oritavancin	8	2	
0147 and oritavancin			0.75 (indifference)
0186	4	1	
Vancomycin	4	1	
0186 and vancomycin			0.5 (synergy)
0186	4	0.25	
Oritavancin	8	2	
0186 and oritavancin			0.3125 (synergy)

a Results represent an average of three to five independent experiments performed in triplicate.

**TABLE 4:** Fractional inhibitory concentration analysis for checkerboard assay^*a*^

Compound	FIC value	Interpretation
0147 and vancomycin	1.06–2	Indifference
0147 and oritavancin	0.56–0.75	Indifference
0186 and vancomycin	2	Indifference
0186 and oritavancin	0.75–2	Indifference
0147 and 0186	1	Indifference

a Results represent three independent experiments performed in triplicate.

## DISCUSSION

We previously identified 0147 as a lead on the basis of its potent antibacterial activity against MSSA, MRSA, and other Gram-positive pathogens as well as its low frequency of spontaneous resistance and increased cardiovascular safety (17). Furthermore, 0147 displayed *in vivo* efficacy against MRSA in a mouse model of septicemia. Compound 0147 targets both S. aureus DNA gyrase (50% inhibitory concentration [IC_50_], 0.22 μM) and topoisomerase IV (IC_50_, 3.5 μM). With DNA gyrase, 0147 induces both single- and double-strand breaks in DNA, with single-strand breaks predominating. Compound 0147 thus serves as a promising lead NBTI. We have also identified another promising NBTI with potent antistaphylococcal activity, 0186 (18). Compound 0186 induces somewhat greater levels of double-strand DNA breaks than 0147 (17). In contrast, the NBTI comparator gepotidacin induces only single-strand breaks (11), thus presenting the opportunity to compare three NBTIs with different biochemical profiles.

In previous studies, the MICs for 0147 and 0186 were comparable for MSSA and MRSA (17, 18). Thus, we opted to use S. aureus ATCC 29213 as our model for this initial study. Compounds 0147 and 0186 exhibited low MICs of 0.125 to 0.25 μg/mL and 0.0625 μg/mL, respectively, against S. aureus ATCC 29213, consistent with our previous studies (17, 18). Both compounds demonstrated bactericidal activity in the MBC assay, as did gepotidacin, and the time-kill assay revealed durable bactericidal activity at 24 h against planktonic S. aureus for all three compounds at 8× MIC. Some regrowth was observed for 0147, 0186, and the comparators gepotidacin and ciprofloxacin, especially at lower concentrations, as has been previously observed (12, 19, 20, 32); however, the mechanism for regrowth is currently unknown (19).

Similar to gepotidacin, 0147 and 0186 yielded low MBEC values, which suggests comparable activity against S. aureus biofilm. This is particularly promising since this is one of the first studies to evaluate the effectiveness of NBTIs in eradicating biofilms. Compound 0186 demonstrated a somewhat higher MBEC-to-MIC ratio (64), suggesting reduced effectiveness toward biofilm compared to planktonic S. aureus. Potential explanations for this observation include reduced penetration of 0186 into biofilm or reduced antibacterial activity against slow-growing/stationary-phase bacteria (26). The MBEC has historically been determined using a variety of methods, for example, growing biofilm on pegs using Innovotech’s biofilm inoculator (33–35). Our MBEC values for oritavancin and vancomycin are consistent with previously published results (24); however, there are variable reports for the MBEC of ciprofloxacin (36). This is likely due to methodological differences.

One strategy that has been suggested for effectively eradicating S. aureus biofilm is combination therapy, more specifically pairing antibiotics with compounds that can weaken or penetrate biofilm (27). The potential benefits of combination therapy include better antibiotic penetration, increased antibiotic efficacy, and suppression of resistance (30, 37). Since NBTIs 0147 and 0186 target DNA gyrase and topoisomerase IV, we used antibiotics that targeted another portion of the bacterial cell or were known to be active against stationary-phase and biofilm S. aureus. Vancomycin, a glycopeptide, is a common antibiotic utilized to treat MRSA infections and has demonstrated promising results in previous combination treatment studies (22, 28–30). Oritavancin, a lipoglycopeptide, effectively eradicates stationary-phase and biofilm S. aureus through its additional mechanism of disrupting membrane integrity (24, 31). As a result, we examined the effects of combining the NBTIs with these glycopeptides to treat S. aureus ATCC 29213 preformed biofilms, a clinically relevant situation. Our results demonstrated synergistic interactions with the combinations of 0147 and vancomycin, 0186 and vancomycin, and 0186 and oritavancin. On the other hand, when testing the same combinations of antibiotics on planktonic S. aureus, the interactions were all indifferent, which has also been observed with gepotidacin (19). These results suggest that the synergistic interactions observed for these combinations are specific to biofilms. Additional studies are needed to determine the mechanism behind this synergistic interaction.

In these studies, we explored the *in vitro* activity of two dioxane-linked NBTIs, 0147 and 0186, against S. aureus 29213. Both compounds demonstrated bactericidal activity against planktonic and biofilm S. aureus ATCC as evidenced by their MIC and MBEC values. Compounds 0147 and 0186 were also shown to prevent biofilm formation at the MIC and/or 2× MIC. While the interactions between the NBTIs and glycopeptides were indifferent against planktonic S. aureus, three combinations (0147 and vancomycin, 0186 and vancomycin, and 0186 and oritavancin) showed synergistic effects against S. aureus biofilm. A limitation of this study is that it employed only a single laboratory strain of S. aureus. Future studies will include testing NBTIs with a USA300 MRSA strain and additional clinical isolates, including from individuals living with cystic fibrosis. An additional limitation was the use of only two approved antibiotic partners in FBIC and checkerboard assays. Future research will investigate additional combinations with mechanistically diverse compounds. Strengths of the current study include the observation of antibacterial activity for NBTIs against biofilms and of synergistic effects between NBTIs and glycopeptides, both of which are novel to the field of NBTIs. Additional studies to assess the efficacy of these compounds using *in vivo* models of infection are ongoing in our laboratories. Ultimately, these results highlight the potential of NBTIs such as 0147 and 0186 as future therapeutics against S. aureus infections.

## MATERIALS AND METHODS

### Bacterial strain.

MSSA laboratory reference strain Staphylococcus aureus ATCC 29213 was utilized throughout these studies. S. aureus ATCC 29213 was grown in cation-adjusted BBL Mueller-Hinton II broth (CAMHB) overnight at 37°C with shaking at 200 rpm.

### Preparation of NBTI compounds and comparator agents.

The synthesis and characterization of 0147 (17) and 0186 (18) have been described previously. Gepotidacin (MedChemExpress), 0147, 0186, and oritavancin diphosphate (Sigma-Aldrich) were dissolved in dimethyl sulfoxide (DMSO) and subsequently diluted in phosphate-buffered saline (PBS) to the designated concentrations. Ciprofloxacin (Fluka Analytical), vancomycin (Novaplus), erythromycin (Sigma-Aldrich), and tetracycline (Fisher BioReagents) were dissolved in water followed by dilution in PBS.

### MIC.

S. aureus ATCC 29213 was grown in CAMHB overnight at 37°C. The MIC of each compound was determined following the broth microdilution protocol set by the Clinical and Laboratory Standards Institute (CLSI) (38). A 50-μL aliquot of overnight culture was seeded into 5 mL of fresh CAMHB, grown to an optical density at 600 nm (OD_600_) of 0.08 to 0.100, and diluted subsequently by a factor of 150 in CAMHB. We incubated 50 μL of the S. aureus ATCC 29213 culture with 50 μL of the compounds over a concentration range of 0.0078 to 8 μg/mL (in serial 2-fold dilutions) overnight at 37°C in a 96-well plate in a humid chamber. The MIC is determined as the lowest concentration of compound where no visible bacterial growth is seen after antibacterial treatment (38). Typical variability for these results is ±1 2-fold dilution. The untreated culture served as the negative control. The results represent three independent experiments performed in triplicate.

### MBCs.

Determination of the MBC was completed after measuring the MIC. A 5-μL aliquot was taken from various wells of the MIC plate and incubated on cation-adjusted BBL Mueller-Hinton II agar (CAMHA) free of antibiotic treatment overnight at 37°C. The MBC is defined as the lowest concentration of test article that fully prevents bacterial growth. Typical variability for these results is ±1 2-fold dilution. Compounds are categorized as bactericidal when the ratio of MBC to MIC is ≤4 and bacteriostatic when the ratio of MBC to MIC is >4 (39, 40). The untreated culture served as the negative control. The results represent three independent experiments performed in triplicate.

### Time-kill assay.

S. aureus ATCC 29213 was grown in CAMHB overnight at 37°C. A 50-μL aliquot of overnight culture was then seeded into 5 mL of fresh CAMHB, grown to an OD_600_ of 0.1, and diluted by a factor of 50 for a starting inoculum of ~10^6^ CFU/mL. The S. aureus ATCC 29213 culture was then treated with the compounds at the MIC and at 8× MIC for 24 h. The untreated culture served as the negative control. Aliquots of the treated culture were serially diluted in PBS and plated on CAMHA at the 0-, 1-, 2-, 4-, 6-, 8-, 12-, and 24-h time points. The CFU were calculated for each time point. Bactericidal concentrations were defined as those achieving a >99.9% (3-log_10_) reduction in CFU/mL compared to the starting inoculum (39). The results represent an average of three independent experiments performed in triplicate.

### Checkerboard assay.

The checkerboard assay was utilized to assess potential synergistic relationships between two antibiotics (e.g., NBTI and glycopeptide). S. aureus ATCC 29213 was grown and prepared as described in the MIC protocol. The compounds were prepared separately through 2-fold serial dilutions in CAMHB in a 96-well plate. Twenty-five microliters of each compound was combined with 50 μL of the S. aureus ATCC 29213 culture. The untreated culture served as the negative control. The 96-well plate was then incubated overnight at 37°C in a humid chamber. The MIC was determined using the Molecular Devices SpectraMax i3x, where no visible growth is equivalent to OD_600_ of <0.07, and confirmed visually (41). We utilized the fractional inhibitory concentration (FIC) equation, $\text{FIC}=\;\frac{\text{MIC}-A_{A+B}}{{\text{MIC}}_{A}}+\frac{\text{MIC}-B_{A+B}}{{\text{MIC}}_{B}}$, to determine potential synergistic relationships between NBTIs and glycopeptides. FIC values of ≤0.5 are labeled “synergy,” FIC values of 0.5 to 4 are labeled “indifference,” and FIC values of >4 are labeled “antagonism” (42–44). The results represent three independent experiments performed in triplicate.

### Inhibition of biofilm formation.

S. aureus ATCC 29213 was grown in Luria broth (LB) with 2% glucose and 2% NaCl overnight at 37°C. The addition of glucose and NaCl to the medium was utilized to promote robust biofilm growth at the bottom of the 96-well plate. A 50-μL aliquot of overnight culture was then seeded into 5 mL of fresh LB with 2% glucose and 2% NaCl, grown to an OD_600_ of 0.1, and diluted by a factor of 10 for a starting CFU of ~10^7^ CFU/mL. The newly prepared culture and treatment were added at the same time to a 96-well plate that was treated with poly-l-lysine, followed by incubation at 37°C in a humid chamber for 24 h. The untreated culture served as the negative control. After incubation, the planktonic culture was removed by aspiration, leaving behind only attached biofilm. The wells were then washed 3× with 150 μL PBS and stained with 0.1% crystal violet (20% ethanol and 80% H_2_O) for 20 min at room temperature. After staining, the crystal violet was removed by aspiration, and the wells were washed 5× with PBS. Crystal violet was then extracted in 33% glacial acetic acid and incubated at room temperature for 25 min. The absorbance was measured using the Molecular Devices SpectraMax i3x at OD_590_ (23). The results represent an average of three or four independent experiments performed in triplicate and were analyzed by one-way analysis of variance (ANOVA) (ns, *P* > 0.05; *, *P* < 0.05; **, *P <* 0.01; ***, *P <* 0.001;****, *P* < 0.0001) using GraphPad Prism version 9.4.1.

### Biofilm eradication assay.

**FIG 4:**
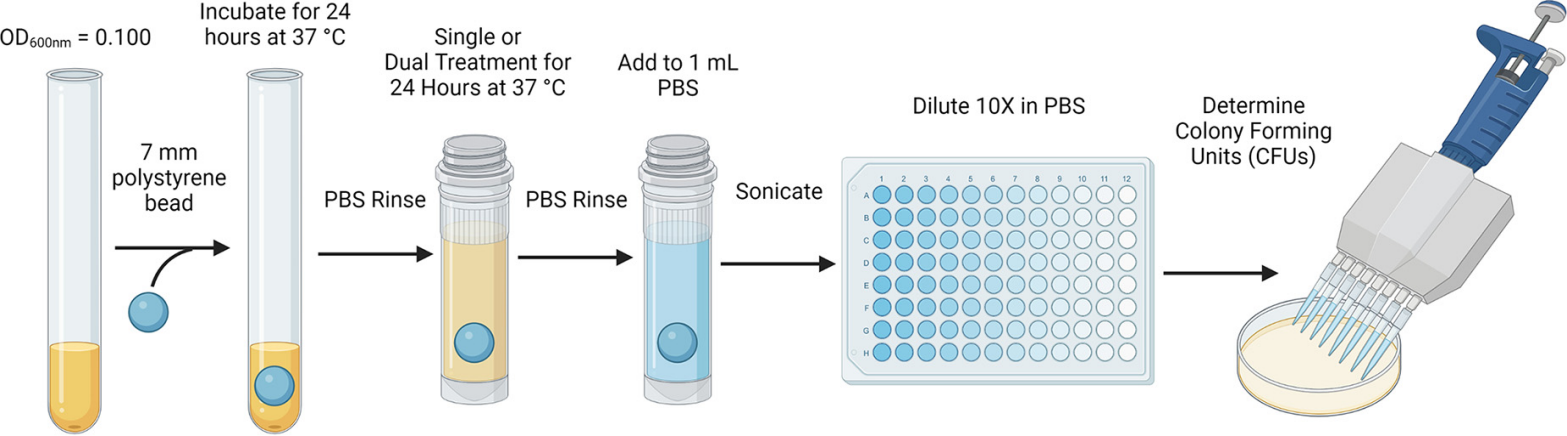
Biofilm bead model for determining the minimum biofilm eradication concentration (MBEC). Created with BioRender.com.

S. aureus ATCC 29213 was grown in CAMHB overnight at 37°C. A 50-μL aliquot of overnight culture was then seeded into 5 mL of fresh CAMHB and grown to an OD_600_ of 0.1. Biofilms were grown on 7-mm polystyrene beads in CAMHB for 24 h at 37°C on a rotator (Fig. 4). Planktonic bacteria were removed from the biofilm by submerging and gently swirling the bead in 1.5 mL of PBS. The beads were then transferred to CAMHB containing the specified test article concentration (single or dual treatment) and incubated for another 24 h at 37°C on a rotator. The beads were washed with 1.5 mL of PBS using the same protocol as previously, followed by sonication into 1 mL of PBS to dislodge the biofilm. CFU of the sonicated samples were enumerated (25). The untreated biofilm bead served as the negative control. The MBEC is defined as the concentration that leads to a >99.9% (3-log_10_) decrease in CFU per milliliter for biofilms (45). Typical variability for these results is ±1 2-fold dilution. The MBEC results represent an average of three to seven independent experiments performed in triplicate. We used the fractional biofilm inhibitory concentration (FBIC) equation and analysis when testing NTBIs and glycopeptides in combination on S. aureus biofilm with the biofilm bead model (46, 47). The FBIC results represent an average of three to five independent experiments performed in triplicate.
